# Comparison of the environmental footprint of the egg industry in the United States in 1960 and 2010^1^

**DOI:** 10.3382/ps.2013-03390

**Published:** 2014-01-21

**Authors:** Nathan Pelletier, Maro Ibarburu, Hongwei Xin

**Affiliations:** * Global Ecologic Environmental Consulting and Management Services, 6200 Silver Star Road, Vernon, BC V1B3P3, Canada; † Egg Industry Center, Iowa State University, 1202 NSRIC, Ames 50011-3310

**Keywords:** egg, pullet, life cycle assessment, environmental footprint, energy return on energy invested

## Abstract

The US egg industry has evolved considerably over recent decades by incorporating new technologies and production practices. To date, there has been no comprehensive assessment of the resource demand and environmental effects of these changes. This study quantifies the environmental footprint of egg production supply chains in the United States for 2010 compared with 1960 using life cycle assessment. The analysis considers changes in both foreground (e.g., hen production performance) and background (e.g., efficiencies of energy provision, fertilizer production, production of feed inputs, and transport modes) system variables. The results revealed that feed efficiency, feed composition, and manure management are the 3 primary factors that determine the environmental impacts of US egg production. Further research and improvements in these areas will aid in continual reduction of the environmental footprint of the US egg industry over time. Per kilogram of eggs produced, the environmental footprint for 2010 is 65% lower in acidifying emissions, 71% lower in eutrophying emissions, 71% lower in greenhouse gas emissions, and 31% lower in cumulative energy demand compared with 1960. Table egg production was 30% higher in 2010; however, the total environmental footprint was 54% lower in acidifying emissions, 63% lower in eutrophying emissions, 63% lower in greenhouse gas emissions, and 13% lower in cumulative energy demand compared with 1960. Reductions in the environmental footprint over the 50-yr interval considered can be attributed to the following: 27 to 30% due to improved efficiencies of background systems, which outweighed the declining energy return on energy invested for primary energy sources; 30 to 44% due to changes in feed composition; and 28 to 43% due to improved bird performance.

## INTRODUCTION

Agricultural production in the United States has advanced considerably over recent decades by incorporating new technologies to make more efficient use of finite resources such as land, water, and energy (Capper et al., 2009; Capper, 2011; Boyd and Cady, 2012; Hamilton et al., 2013). Egg production has followed a similar trend, achieving productivity levels that would have been difficult to imagine half a century ago. To date, there has been no comprehensive assessment of the resource demand and environmental effects of these changes in production practices and efficiencies.

Life cycle assessment (**LCA**) is an analytical framework for characterizing material and energy flows and emissions along product supply chains and for quantifying how these contribute to a variety of resource use, human health, and environmental impact potentials. The methodology has been standardized by the International Organization for Standardization (**ISO**) in the ISO 14040–14044 standard series (ISO, 2006). The key strength of LCA is that it facilitates identification of opportunities for mitigating key drivers of impacts while being sensitive to burden-shifting, whether between supply chain stages or between different kinds of environmental impacts (for example, greenhouse gas emissions versus ozone-depleting emissions).

In this study, we applied ISO 14044-compliant LCA methods (ISO, 2006) to quantify the changes in the environmental footprint of US egg production between 1960 and 2010. The specific objectives of the study were to

1. develop models of US egg production supply chains for 1960 and 2010 with regard to both foreground system variables (such as feed conversion or efficiency, bird body weight, bird mortality rate, hen-day egg production) and background system variables (such as efficiencies of energy provision, fertilizer production, production of feed inputs, transport modes);
2. characterize supply chain environmental performance of the US egg industry for 1960 and 2010 in terms of the following:
  - cumulative energy demand (**CED**, expressed in MJ)—all embodied renewable and nonrenewable energy inputs,
  - acidifying emissions (expressed in SO_2_-equivalent units)—all emissions that contribute to ecosystem acidification,
  - eutrophying emissions (expressed in PO_4_-equivalent units)—all emissions of N- and P-containing compounds that contribute to eutrophication of fresh water bodies, and
  - greenhouse gas (**GHG**) emissions (expressed in CO_2_-equivalent units)—all emissions that contribute to increased atmospheric radiative forcing;
3. determine the magnitude of changes in production performance and environmental impacts associated with technological and management advancements over the 50-yr interval.

The results of the study are intended to provide the US egg industry and other stakeholders with science-based information concerning the impact of advances in egg production on resource utilization efficiencies and environmental performance. The study also offers insight into areas for further mitigation of environmental impacts and conservation of natural resources.

## MATERIALS AND METHODS

### Goal and Scope

**Figure 1.**
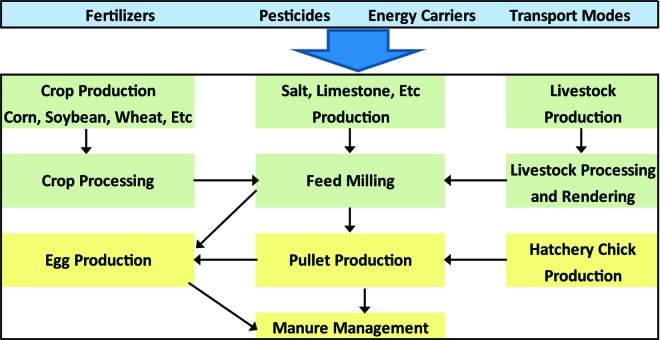
System boundaries for a life cycle assessment of egg production in the United States for 1960 and 2010 (background processes such as fertilizers, pesticides, and transport modes were derived from the EcoInvent (2010) database but were modified to reflect US energy carriers). Color version available in the online PDF.

The system boundaries for this analysis included all direct and indirect inputs and emissions arising from the production of raw materials for feed inputs, feed input processing, feed milling, hatcheries, and farm-level material and energy use at pullet and layer facilities for both 1960 and 2010 (Figure 1). The production and maintenance of infrastructure such as machinery and buildings were not included because, in high production-volume contexts, their contributions are typically trivial (Ayer and Tyedmers, 2009). These parallel models were subsequently used to evaluate the environmental footprint of US egg production in terms of CED, GHG, and acidifying and eutrophying emissions for 1960 versus 2010.

### Life Cycle Inventory: 2010 Model

Foreground system data refer to information unique to the product system of interest. Foreground system data for feed milling, pullet, and layer facilities were collected via anonymous surveys from participating companies. The data collected represented 57.1 million pullets and 92.5 million laying hens, accounting for 26% of pullet stock and 33% of laying-hen stock in the United States in 2010. In the absence of company-specific information for hatcheries (no participants in the study), data for hatcheries were adopted from an earlier study of US broiler production systems (Pelletier, 2008).

Background system data refer to information regarding processes linked to the foreground system in the supply chain of interest, but shared with other supply chains. In the context of our analysis, this included the provision of energy carriers (i.e., energy sources such as fossil fuels and electricity), inputs to crop production and other feed input production and processing systems, and transportation modes. Background system data for the production and processing of feed ingredients were adapted from recent LCA studies of beef and pork production supply chains in the Upper Midwestern United States (Pelletier et al., 2010a,b) and global salmon aquaculture supply chains (Pelletier et al., 2009). These studies used identical modeling parameters to those of the current analysis and hence the feed input models could be directly adopted. Other background system data, including the provision of energy carriers, fertilizers, pesticides, and transportation models, were derived from the EcoInvent (2010) database and modified to reflect US energy inputs.

#### Modeling N and P Emissions.

Nitrogen and P emissions from pullet and layer facilities were calculated using a nutrient balance model based on feed composition and assuming that hen body mass contains 2.2% N and 0.6% P, and eggs contain 1.7% N and 0.21% P as reported by Koelsch (2007). Nitrogen excretion estimates were used to calculate direct nitrous oxide, ammonia, and nitric oxide emissions from manure management and indirect nitrous oxide emissions from nitrate leaching and ammonia emissions following IPCC (2006) protocols and relevant Tier I and Tier II emission factors at time of deposition, storage, and application. Methane emissions from manure management were calculated following IPCC (2006) Tier I protocols. Phosphorus emissions were calculated at a 2.9% leaching rate at time of application of manure to agricultural lands following Dalgaard et al. (2008).

#### Coproduct Allocation.

Coproduct allocation is required to apportion resource use and emissions between the products of multi-output systems. The mass-adjusted gross chemical energy content of coproducts was used as the basis for all allocation decisions because (1) producing caloric energy is the root driver of all food production activities, and (2) the chemical energy of food products present in raw materials is apportioned between processed outputs in a quantifiable manner that speaks directly to the ecological efficiency with which the system provides available food energy (whether for direct human consumption or for livestock feed). This allocation strategy is consistent with the ISO 14044 specification that the “inventory is based on material balances between inputs and outputs. Allocation procedures should therefore approximate as much as possible such fundamental input/output relationships and characteristics” (ISO, 2006). A detailed discussion of this allocation rationale was given by Pelletier and Tyedmers (2011). This approach was chosen over economic allocation, which is sometimes used in reported food system LCA, because (1) economic allocation is a last-resort option in the ISO 14044 hierarchy (ISO, 2006) and (2) the use of economic allocation typically produces results that poorly reflect the physical reality of the systems modeled (Pelletier and Tyedmers, 2011). The use of substitution (following a consequential data modeling approach) was similarly deemed inappropriate for our analysis, which was intended to establish baseline models rather than to model market-level consequences of possible changes in production systems.

### Life Cycle Inventory: 1960 Model

In developing a model to represent average US egg supply chain characteristics in 1960, industry and academic expert sources were consulted and published literature referenced. This required estimating performance efficiencies for both foreground and background system variables. Where we were not able to identify a robust basis for characterizing specific foreground system variables for 1960 (e.g., energy use in poultry housing systems), we used 2010 data in proxy, but with modifications to accommodate our 1960s background system variables. This likely resulted in an underestimation of differences in the environmental performance of egg production in 1960 versus 2010. Modeling of key variables for 1960 is described below.

#### 1960 Energy Carrier Models.

Energy return on energy invested (**EROI**) is a measure of the energy efficiency of energy production. It indicates the amount of energy yield for a given energy carrier (e.g., oil, gas, coal, or electricity) relative to the energy input to its procurement. Several researchers have reported declining EROI values for different energy carriers over time (Gangon et al., 2009; Guilford et al., 2011; Lambert et al., 2012). This is because, as easily accessible, high-quality energy resources are exhausted, an increasing proportion of energy production derives from less-accessible, marginal energy resources that are more energy-intensive to exploit. In short, over time, more energy is required to produce an equivalent unit of energy. From a life cycle perspective, taking into account this changing efficiency and the associated changes in environmental burdens is essential to realistic, time-sensitive modeling.

The EROI values at any given time differ between energy carriers, region of production, and production technology. Moreover, EROI can be described from both production and consumption perspectives. Because energy commodities are widely traded, calculating EROI values for energy carriers used in a given country requires attention to trade patterns and, in the case of electricity, country-specific energy mixes.

For the purpose of the present analysis, EROI values for the United States as well as global EROI values for the production of specific energy carriers were adopted from or calculated based on the work of Gangon et al. (2009), Guilford et al. (2011), and Lambert et al. (2012). In turn, these were used to calculate EROI for primary energy carriers used in the United States in 1960 and 2010 using US Energy Information Administration (**USEIA**; 2012) statistics for US consumption and imports of energy products. The USEIA (2012) statistics for the energy mixes used in US electricity production were also employed to calculate 1960 and 2010 EROI values for electricity. On this basis, scaling factors were derived to represent the comparative EROI of energy carriers between 1960 and 2010 (Table 1). These factors were applied to modify the life cycle inventories used for 2010 energy carriers (adapted from the EcoInvent database) to arrive at 1960 energy carrier life cycle inventories that approximate changes in the environmental performance profile of energy carriers used in the United States over this interval. Potential differences in distribution losses for electricity (grid efficiencies) in 1960 compared with 2010 were not considered.

**Table 1.** Estimated energy return on energy invested values for energy carriers used in 1960 and 2010 in the United States

Energy carrier	1960	2010	Scaling factor between 2010 and 1960
Coal	75	60	0.8
Oil/gas	47	15	0.3
Nuclear and renewables	15	15	1.0
Electricity	14	14	1.0

#### 1960 Fertilizer Production Models.

The US fertilizer mixes for 1960 were derived from International Fertilizer Industry Association (**IFA**) statistics (IFA, 2012). Ammonia production accounts for 87% of the fertilizer industry's energy consumption (IFA, 2009). Based on data regarding improvements in the efficiency of ammonia plants over time, IFA (2009) shows that efficiencies improved from 58 to 28 MJ of energy required per metric ton of ammonia produced between 1960 and 2010. Effectively, this means that producing ammonia in 1960 required 2.07 times as much direct energy input as in 2010. This ratio was hence applied to scale the energy inputs for average ammonia production for the EcoInvent (2010) life cycle inventory used to represent contemporary ammonia production to arrive at a representative 1960 life cycle inventory.

For all other fertilizer “building blocks,” Kongshaug (1998) provides estimates of net energy consumption for “old technology–1970,” “average technology–1998,” and “best available technology–1998.” These estimates largely distinguish between net energy production in the form of steam, which may or may not be productively used. The modified EcoInvent processes for fertilizer production (originally representing average EU production, but modified to reflect US energy inputs) used in the present analysis assume that net energy produced is lost as waste heat. For the purpose of the current analysis, this assumption was similarly adopted; namely, we did not distinguish between sulfuric acid, nitric acid, and phosphoric acid net energy production in 1960 versus 2010 (although we did apply the modified energy carrier inventories in the 1960 fertilizer production models).

#### 1960 Freight Transport Models.

United States Department of Energy (**US-DOE**) data were used to calculate differences in the energy efficiency of freight transport by mode in 1960 compared with 2010 (US-DOE, 2012). The energy intensity of US heavy truck freight decreased from 24,960 BTU (26.3 MJ) per vehicle mile in 1970 to 21,463 BTU (22.6 MJ) per vehicle mile in 2010, with an average annual decrease of 0.4%. Making a linear extrapolation to 1960 on this basis, estimated energy intensity of road freight was 25,977 BTU (27.4 MJ) per vehicle mile. A correction factor of 1.21 was therefore applied to the EcoInvent (2010) model used to represent US road freight energy use in 2010 for the 1960 model.

The energy intensity of US rail freight decreased from 691 BTU (0.73 MJ) per ton-mile in 1970 to 289 BTU (0.30 MJ) per ton-mile in 2010, with an average annual decrease of 2.2% (US-DOE, 2012). Making a linear extrapolation to 1960 on this basis, estimated energy intensity of US rail freight was 859 BTU (0.91 MJ) per ton-mile. A correction factor of 2.97 was therefore applied to the EcoInvent (2010) model used to represent US rail freight energy use in 2010 for the 1960 model.

The US-DOE (2012) only provides data for changes in the energy intensity of water freight on taxable waterways from 1997 (266 BTU or 0.28 MJ per ton-mile) to 2010 (217 BTU or 0.23 MJ per ton-mile), with an average annual decrease of 2.20%. Extrapolating back to 1960 suggests an energy intensity of 595 BTU (0.63 MJ) per ton-mile in 1960, which would imply a correction factor of 2.74. This is very similar to the estimated correction factor for rail freight extrapolating from 1970 to 2010 time series data. This estimate was the weakest, however, given that efficiency in 1960 was extrapolated from only 14 yr of data spanning 1997 to 2010.

For comparison, using data from Fearnley's Review (2012) for world seaborne trade from 1969 to 2010 and estimates of marine fuel use from 1950 to 2010 (Eyring et al.*,* 2005), the estimated correction factor for global ocean freight was 1.33. Elsewhere, a study by Lloyd's Register (2008) suggested a 75% improvement in fuel efficiency for shipping between 1976 and 2007. However, for consistency with our calculations for road and rail freight, we adopted the correction factor of 2.74.

#### 1960 Feed Input Models.

Smil et al. (1983) reported energy inputs to US corn production for 1959. On this basis, direct energy inputs had declined 61% per unit production compared with reported energy inputs to corn production in 2001 (adopted for 2010) as estimated by the US National Agricultural Statistics Service (NASS, 2004). No similar estimates were available for our 1960s models for soy or wheat; hence, a proportionate decline was assumed in energy inputs relative to NASS (2004) energy use estimates for soybeans in 2002 and wheat in 1998. Pesticide use for crops was based on statistics for 1964 provided by the USDA (1995). Fertilizer use was also based on statistics for 1964 provided by USDA (2012). Sulfur and lime inputs were assumed to be similar between 1960 and 2010. Crop yield data for 1960 were taken from the USDA Feed Grains Database and USDA Oil Seeds Database.

All animal-derived and other feed inputs were based on the LCA models reported by Pelletier et al. (2009; for fish meal) and Pelletier et al. (2010a,b; for porcine and ruminant materials), which were created using identical modeling protocols to those used for the 2010 model in the current analysis. For ruminant production, the model of Pelletier et al. was used for grass-fed beef production to represent 1960s conditions (versus their model of conventional, feedlot production to represent 2010 conditions). For pig production, the model of Pelletier et al. was used for low-performance niche production to approximate 1960s conditions (versus their model of conventional, commodity production to represent 2010 conditions). In the absence of an alternative model for broiler chicken production (most common source of processing coproducts rendered into poultry by-product meal and fat), it was assumed that the spent hens destined for rendering as modeled in the current analysis were used for the production of poultry by-product meal and fat.

#### 1960 Pullet and Layer Production Models.

Bird performance data for pullet and layer production were taken from Winter and Funk (1960), and verified with industry and academia experts. For pullets, this included feed composition, feed consumed per pullet sold, mortality rate (% of initial placement), and age and BW of pullets at the time of moving into the layer houses. For layers, this included feed composition, daily feed consumption, annual egg production per hen, egg weight, feed conversion, mortality rate, and number of pullets added to layer houses per year.

### Life Cycle Impact Assessment and Interpretation

Impact assessment in LCA involves calculating the contributions made by the material and energy inputs and outputs tabulated in the inventory phase to a specified suite of environmental impact categories. In this study, CED and GHG acidifying and eutrophying emissions were quantified. Cumulative energy demand (MJ) accounts for conversion efficiencies and the quality of energy inputs (Frischknecht et al., 2007). Quantification of GHG emissions (CO_2_-equivalency over a 100-yr time horizon according to IPCC, 2006), acidifying emissions (SO_2_-equivalency), and eutrophying emissions (PO_4_-equivalency) followed the CML 2 Baseline 2000 method (Guinee et al., 2001).

The environmental impacts were first assessed for each supply chain node considered, then for supply chains in aggregate. Results for the 1960 and 2010 models were subsequently compared to determine differences in environmental performance over time. More detailed contribution analyses were conducted to determine the extent to which the observed differences in environmental performance between 1960 and 2010 were attributable to various factors or model assumptions. The first such analysis evaluated the influence of differences in background system variables only between 1960 and 2010 (i.e., production efficiencies for energy carriers, fertilizers, transport modes, and feed inputs). Here, all 1960 submodels were replaced with 2010 submodels for these parameters. The second analysis used the same feed composition as 2010 in the 1960 model, and also replaced all 1960s background system submodels with 2010 submodels to determine the differences strictly attributable to changes in either feed composition or animal husbandry practices and performance over time.

## RESULTS AND DISCUSSION

### Life Cycle Inventory Results

The life cycle inventory data used for the 2010 and 1960 models of US egg production supply chains are presented in Tables 2 to 9. Inventory data for production and processing of individual feed ingredients (other than corn, wheat, and soy) are not provided herein but can be found in Pelletier et al. (2009, 2010a,b).

**Table 2.** Life cycle inventory data per metric ton of corn, soy, and wheat produced in the United States in 1960 and 2010

	2010				1960		
Item	Corn	Soy	Wheat	Corn	Soy	Wheat
Input						
Fertilizer (kg)						
N	16.1	1.12	20.1	16.6	0.74	9.17
P_2_O_5_	5.55	5.53	6.91	10.8	2.72	7.03
K_2_O	5.71	7.75	1.36	8.50	3.35	3.93
Sulfur	0.27	0.13	0.53	0.27	0.13	0.53
Lime	33.5	0.00	0.00	33.4	0.00	0.00
Energy						
Diesel (L)	4.49	10.9	13.2	4.47	17.5	21.3
Gas (L)	1.17	3.49	3.02	12.1	5.62	4.86
Liquid propane gas (L)	7.02	0.00	3.82	2.68	0.00	6.16
Electricity (kWh)	4.33	0.00	11.9	0.00	0.00	19.19
Total pesticides (kg)	0.25	0.46	0.29	0.20	0.21	0.12
Herbicides	0.24	0.45	0.12	0.13	0.09	0.11
Insecticides	0.01	0.01	0.00	0.08	0.11	0.01
Other (fungicides)	0.00	0.00	0.17	0.00	0.00	0.00
Seed (kg)	2.10	23.4	34.5	20.5	45.0	41.8
Output						
Nitrous oxide (kg)	0.46	0.25	0.55	0.49	0.27	0.36
Ammonia (kg)	2.38	2.19	4.13	3.57	3.91	4.46
Nitric oxide (kg)	0.35	0.02	0.43	0.36	0.02	0.20
Carbon dioxide (kg)	17.2	0.17	3.04	14.3	0.03	0.42
Nitrate (kg)	1.44	0.00	0.00	4.49	0.00	0.00
Phosphate (kg)	0.00	0.00	0.03	0.14	0.00	0.00
Yield (t)	1.00	1.00	1.00	1.00	1.00	1.00

Substantial increases in crop yield over the 50 yr, in many cases, offset the increases in resource inputs, with some inputs higher per unit yield in 1960 or in 2010, depending on the input and crop (Table 2). For feed milling in 2010, the reported proportions and total amounts of different energy carrier inputs per metric ton of feed milled were highly variable (Table 3), as were the distances traveled for the feed inputs sourced (Table 4). For the purpose of our analysis, total consumption-weighted averages were used to arrive at the proportions and feed transport distances modeled.

**Table 3.** Energy inputs per metric ton (1,000 kg or 2,200 lb) of pullet/layer feed milled in reporting facilities in the United States in 2010 (representing a total production of 2,679,405 t of feed)^1^

Item	Production- weighted average	Range
Electricity (MJ)	15.8	1.8–52.9
Diesel (MJ)	51.1	0–122.8
Gasoline (MJ)	1.5	0–3.4
Natural gas (MJ)	0	0–0.02

^1^This data set was also used for the 1960 model.

**Table 4.** Distances traveled for inputs to pullet/layer feed milled in reporting facilities in the United States in 2010 (representing a total production of 2,679,405 t)^1^

Feed input	Distance to processor^2^ (km)	Distance to feed mill^3^ (km)	Range
Corn		27	24–48
Corn dried distillers grains with solubles	25	116	1–193
Soy meal	100	96	29–133
Bakery material	wheat: 100 to flour mill	258	97–587
	flour: 1,000 to bakery		
Wheat middlings	100	474	241–604
Meat and bone meal	100	151	56–322
Fat	100	272	0–579
Salt	25	370	0–861
Limestone	100	142	0–241
Calcium	100	186	137–225
Phosphate	100	239	0–861
Trace vitamins	100	325	0–563

^1^This data set was also used for the 1960 model.

^2^Assumed average distances.

^3^Production-weighted average.

Reported data were similarly variable for pullet and layer facilities for parameters such as water use, energy use, manure mass, and so on. Again, although the ranges of values are reported in the proceeding tables, production-weighted averages were used to construct the life cycle inventory model.

Both the types and inclusion rate of ingredients in pullet and layer feeds changed between 1960 and 2010 (Tables 5 and 6). Whereas corn and soy products constituted the core bulk ingredients for both periods, wheat was a more important input in 1960 (10% wheat middlings in layer diets vs. 0.8% in 2010). Several ingredients were also used in only one period or the other, for example, green feed (modeled here as alfalfa) and fish meal in 1960 pullet feeds, and bakery material in 2010 pullet and layer feeds. Notable here is the reduced fraction of animal-derived materials (approximately 50% of 1960 levels) in contemporary feeds. The N and P contents of different feed ingredients, as used to estimate nutrient balances, are listed in Table 7.

**Table 5.** Pullet feed composition for egg production in the United States in 1960 (based on Winter and Funk, 1960) and 2010 (based on the production-weighted average of feed composition data from reporting pullet producers)

Item	1960 (% inclusion)	2010 (% inclusion)	2010 (range)
Corn	78.1	60.0	41.0–70.7
Corn dried distillers grains with solubles	1.0	6.2	0–13.0
Soy meal	10.3	21.0	13.0–27.0
Dehydrated green feed^1^	3.0	0.0	^ ^N/A^2^
Fish meal	1.2	0.0	N/A
Bakery material	0.0	1.0	0–13.0
Wheat middlings	0.0	0.9	0–7.0
Meat and bone meal^3^	2.5	1.0	0–5.7
Fat^4^	0.3	0.9	0–1.7
Salt	0.5	0.3	0–0.4
Limestone	1.5	6.2	0–10.5
Dicalcium phosphate	0.6	0.0	N/A
Calcium	0.0	1.3	0–10.0
Phosphate	0.0	0.7	0–1.5
Other^5^	1.0	0.5	0–2.1

^1^Modeled as alfalfa hay based on Pelletier et al. (2010a).

^2^N/A = not applicable.

^3^63% ruminant, 26% porcine, 11% poultry (assumed same as 2010).

^4^50% poultry, 50% vegetable (assumed to be soy oil; assumed same as 2010).

^5^Includes trace vitamins and minerals, modeled as dl-methionine.

**Table 6.** Layer feed composition for egg production in the United States in 1960 (based on Winter and Funk, 1960) and in 2010 (based on feed composition data from reporting egg producers)

Item	1960 (% inclusion)	2010 (% inclusion)	2010 (range)
Corn	63.9	58.6	40.5–69.2
Corn dried distillers grains with solubles	0	6.1	0–15.1
Soy meal	12	19.3	10.0–25.7
Bakery material		0.9	0–12.4
Wheat middlings	10	0.8	0–9.9
Dehydrated green feed^1^	2.5	0	N/A^2^
Meat and bone meal^3^	5	1.8	0–7.8
Fat^4^	1	0.9	0–4.4
Salt	0.5	0.3	0–1.0
Limestone	3.7	6.8	0–11.6
Dicalcium phosphate	1.3	0	N/A
Calcium	0	2.1	0–9.8
Phosphate	0	0.5	0–1.0
Other^5^	0.1	0.5	0–1.8

^1^Modeled as alfalfa hay.

^2^N/A = not applicable.

^3^81% ruminant, 17% porcine, 2% poultry.

^4^4% ruminant, 2% porcine, 58.5% poultry, 35.5% vegetable (assumed to be soy oil).

^5^Includes trace vitamins and minerals, modeled as dl-methionine.

**Table 7.** Proximate composition of feed inputs used for calculating intake, excretion, and losses of N and P

Feed ingredient	% N	% P
Corn	1.224	0.260
Corn dried distillers grains with solubles	4.224	0.710
Soybean meal	6.899	0.620
Bakery by-product	1.728	0.250
Wheat middlings	2.706	0.910
Alfalfa hay (17% CP)	2.720	0.250
Meat and bone meal	8.000	4.000
Fish meal (66% CP)	10.56	3.150
Fat	0	0
Limestone	0	0.020
Phosphate	0	0.4364
Trace vitamins	0	0
Methionine	8.750	0

Perhaps most striking at the inventory level were the differences in resources consumed and other performance parameters for pullet (Table 8) and layer (Table 9) production in 1960 compared with 2010. Feed consumption per pullet raised decreased by 48% over the 50-yr interval, in part explained by a 30% lower BW at the onset of production and in part by a 70% lower mortality rate (Table 8). As a result of reduced mortality, the number of chicks required (per thousand pullets produced) also decreased by a net 8.6% (Table 8). At the same time, estimated losses of N and P decreased by 39 and 60%, respectively. Unfortunately, data for energy inputs to pullet facilities in 1960 could not be found; hence, they were assumed comparable to 2010.

**Table 8.** Life cycle inventory data for the production of 1,000 pullets in the United States in 1960 (based on Winter and Funk, 1960) and in 2010 (based on the production-weighted average data from reporting pullet producers representing 57,116,182 pullets)

Item	1960 average	2010 average	2010 range	Percent change
Chicks	1,133	1,036	1,021–1,047	−9
Mass/chick (g)	39.8	39.8	39.1–40.0	0
Distance (km)	434	434	32.2–845	0
Feed (kg)	10.2	5.27	4.31–5.75	−48
Distance (km)	19.2	19.2	0–112	0
Water^1^ (m^3^)	17.9	9.22	7.54–10.1	−48
Energy^2^ (MJ)				
Electricity	3,015	3,015	1,425–5,721	0
Diesel	105	105	0–1,084	0
Gasoline	95.8	95.8	0–517	0
Propane	1,654	1,654	0–4,747	0
Natural gas	187	187	0–1,932	0
Fuel oil	2.63	2.63	0–158	0
Output				
Pullets	1,000	1,000	1,000	0
Mass (t)	1.74	1.22	1.16–1.30	−30
Manure^3^ (t)	6.46	3.38	0.59–4.59	−48
Distance^4^ (km)	10.0	10.0		0
Estimated N loss (kg)	178	108	81.9–122	−39
Estimated P loss (kg)	32.9	13.3	9.09–15.7	−60
BW (kg/bird)	1.7	1.2	1.16–1.30	−30
Mortality rate (%)	11.7	3.5	2.1–4.7	−70

^1^Water use estimated as 1.75 × feed input.

^2^Year 1960 data assumed to be same as 2010.

^3^Manure mass on an as-removed basis, assuming proportionate to the ratio of feed use to manure production in 2010.

^4^Assumed distance of travel from farm to destination of manure application.

For egg production, lower bird BW (2.04 kg/hen in 1960 vs. 1.54 kg/hen in 2010) was one of the main drivers for the observed 26% lower feed consumption per hen in 2010 (Table 9). Lower daily feed use, combined with a 27% higher hen-day egg production and a 57% lower mortality rate, resulted in 42% less feed consumed per kilogram of egg produced. The number of pullets sourced per metric ton of eggs produced also decreased by 22% (Table 9) due to lower mortality. Nitrogen and P emissions decreased by 47 and 64%, respectively.

### Interpretation of Life Cycle Impact Assessment Results

#### Life Cycle Impact Assessment Results for Energy Carriers in 1960 Versus 2010.

**Figure 2.**
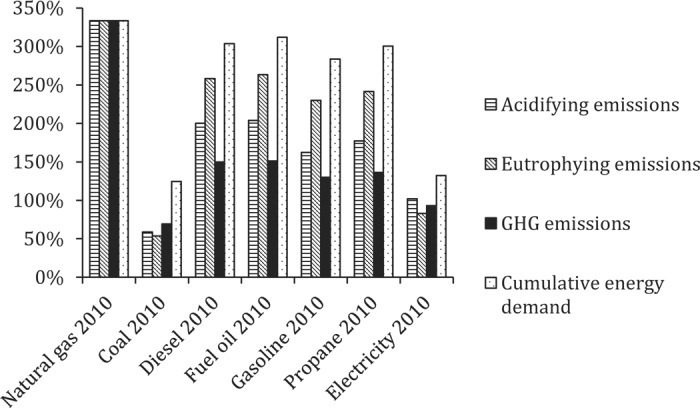
Life cycle impact assessment results for energy carriers used in the United States in 2010 compared with 1960 (all impacts for 2010 presented as a percentage of impacts in 1960). GHG = greenhouse gas.

Energy return on energy invested was substantially higher in 1960 for all primary energy carriers other than coal. As a result, CED and emissions were correspondingly higher in 2010 (Figure 2). The smaller difference for coal in 1960 is explained by the low energy costs of extracting coal relative to the energy costs of transporting coal to markets. Because rail and water freight transport modes were considerably less energy efficient in 1960, these differences effectively offset differences in EROI for coal in 1960 compared with 2010. Eutrophying and GHG emissions for electricity production were also slightly higher in 1960 (Figure 2), largely due to 2 factors. The first factor is the higher fraction of (in particular) coal and other fossil fuels in the 1960 energy mix compared with a greater share of nuclear power generation in 2010. The second factor is the lower efficiency of transforming primary energy carriers into electricity in 1960.

#### Life Cycle Impact Assessment Results for Fertilizer Inputs in 1960 Versus 2010.

**Figure 3.**
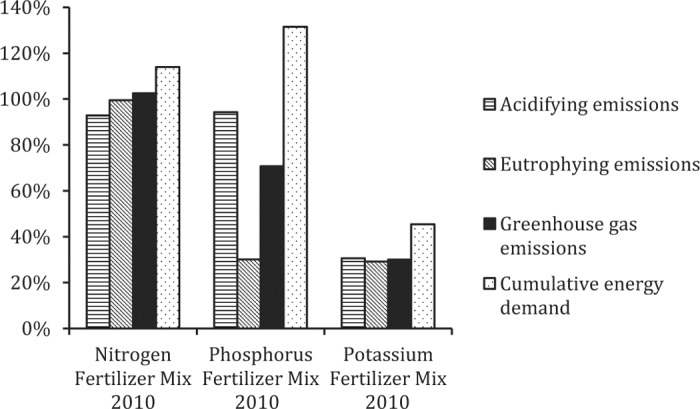
Life cycle impact assessment results for average US fertilizer mixes in 2010 compared with 1960 (all impacts for 2010 presented as a percentage of impacts in 1960).

Despite the substantial increases in the energy efficiency of ammonia production, declining EROI values for energy production effectively offset these gains. As a result, the comparative impacts of N fertilizers consumed in the United States in 2010 were very similar to those of 1960. Impacts for P fertilizer were also similar, with the exception of considerably higher eutrophication impacts in 1960, mostly due to the larger fraction of triple super phosphate in the 1960 fertilizer mix. In contrast, all impacts associated with the US potassium fertilizer mix were substantially higher in 1960 compared with 2010 due to the predominance of more energy-intensive K sources in 1960 versus greater reliance on less energy-intensive potassium chloride in 2010 (Figure 3).

#### Life Cycle Impact Assessment Results for Transport Modes in 1960 Versus 2010.

**Figure 4.**
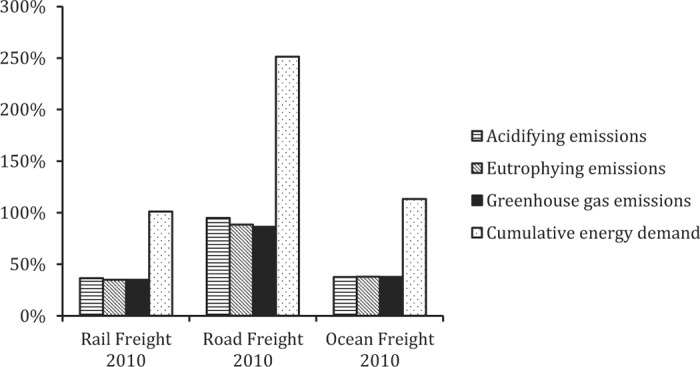
Life cycle impact assessment results per metric ton-kilometer for ocean, rail, and road freight in the United States in 2010 compared with 1960 (all impacts for 2010 presented as a percentage of impacts in 1960).

Acidifying, eutrophying, and GHG emissions per metric ton-kilometer of freight transport were considerably higher in 1960 compared with 2010 for both rail and ocean freight. Interestingly, the declining EROI of fossils fuels over this interval offset almost exactly the improved fuel efficiencies enjoyed by contemporary rail and ocean freight, resulting in very similar CED. For road freight, in contrast, CED was much lower in 1960, and all other impacts very similar to those estimated for 2010. This outcome reflects the lower efficiency gains for road freight compared with rail and ocean freight for the 50-yr interval (Figure 4).

#### Life Cycle Impact Assessment Results for Feed Inputs in 1960 Versus 2010.

In general, the production of raw materials was the largest contributor to impacts for feed inputs to pullet and layer systems, although processing-related emissions were notable for some inputs such as corn dried distillers grains with solubles. Milling-related impacts accounted for a very small fraction of emissions per metric ton of feed produced. Production of animal-derived feed inputs was most impactful across the impact categories. This is unsurprising given the nature of feed conversion, which effectively acts as a multiplier for the impacts of producing the underpinning feed inputs, along with other inputs to animal husbandry, processing, and reduction of processing coproducts into meals and fats. This is particularly true for the production of meat and bone meal and fat from ruminant sources compared with porcine and poultry sources because feed inputs and associated emissions to produce ruminants are considerably higher.

**Figure 5.**
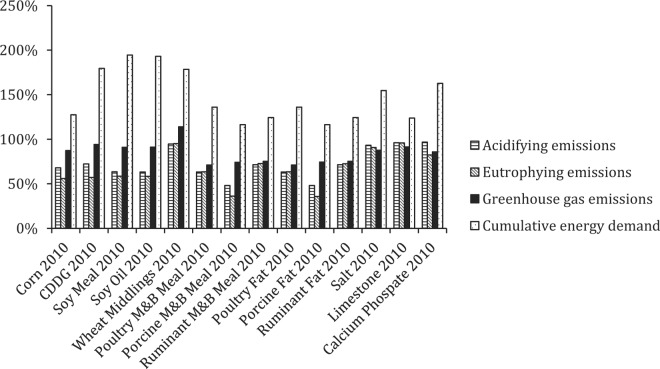
Life cycle impact assessment results for feed inputs to US pullet and layer systems (at the farm or processor gate) in 2010 compared with 1960 (all impacts for 2010 presented as a percentage of impacts in 1960). CDDG = corn dried distillers grains. M+B Meal = meat and bone meal.

Emission-related impacts for feed inputs produced in 1960 were almost universally higher than those in 2010. This reflected a combination of factors, including improved efficiencies of N fertilizer production, transport modes and, in particular, much-improved yields in 2010. The opposite was true for CED, however, where declining EROI effectively outweighed other efficiency gains (Table 10, Figure 5).

**Table 9.** Life cycle inventory data per metric ton of eggs produced in the United States in 1960 (based on Winter and Funk, 1960) and in 2010 (based on the production-weighted average data from reporting egg producers representing 1,542,507.6 t of eggs)

Item	1960 average	2010 average	2010 range	Percent change
Pullets	46	36	21–50	−22
Distance (km)	52.9	52.9	1.61–452	0
Layer feed consumption				
kg/100 layers per d	12.23	9.03	8.1–11.3	−26
kg of feed/kg of eggs	3.44	1.98	1.76–2.32	−42
Distance (km)	12.6	12.6	0–53.1	0
Water (m^3^)	6.25	4.26	3.06–6.58	−32
Energy^1^ (MJ)				
Electricity	557	557	335–1,030	0
Diesel	69	69	0–318	0
Gasoline	9	9	0–34.0	0
Natural gas	4	4	0–102	0
Liquid propane gas	81	81	0–634	0
Output				
Egg production (t)	1	1	1	0
Eggs/100 layers per d	59.18	75.34	68.8–81.1	27
Eggs/layer per yr	216	275	251–296	27
Mass/egg (g)	60.5	60.0	54–63	−1
Spent hens^2^				
Mass (kg)	64.4	50	32.0–70.0	−22
Distance (km)	100	100	100	0
Manure hauled^3^ (kg)	1,980	1,140	510–2,350	−42
Distance^4^ (km)	14.4	14.4	0–32.2	0
Estimated N loss (kg)	61.7	32.4	32.4–45.3	−47
Estimated P loss (kg)	16.1	5.78	9.23–9.87	−64
Mortality^5^				
Rate (% per yr)	15.8	6.7	1.2–8.4	−57
Mass (kg)	11.6	5.47	1.10–11.0	−53

^1^Year 1960 data assumed same as 2010.

^2^34.5% to human consumption, 4.5% to pet food, 49.4% to rendering, 6.2% to composting, 5.0% to other.

^3^Manure mass at time of removal. Moisture content varies, depending on residency time and management strategy.

^4^Estimated distance at removed mass.

^5^Includes culls; 60.3% to rendering, 25.2% to composting, 0.5% to burial, 2.1% to landfill, 11.8% to incineration (assuming no energy recovery).

**Table 10.** Life cycle impact assessment results for acidifying emissions, eutrophying emissions, greenhouse gas (GHG) emissions, and cumulative energy demand (CED) per metric ton of feed inputs at the farm/processor gate in the United States in 1960 and 2010^1^

Feed ingredient	Year	Acidifying emissions (kg of SO_2_-e)	Eutrophying emissions (kg of PO_4_-e)	GHG emissions (kg of CO_2_-e)	CED (MJ)
Corn	1960	7	2	345	1,380
	2010	5	1	301	1,759
CDDGS	1960	10	2	764	4,425
	2010	7	1	719	7,949
Soy meal	1960	7	1	249	1,337
	2010	4	1	227	2,601
Soy oil	1960	15	3	541	2,909
	2010	9	2	493	5,621
Bakery material	1960				
	2010	8	2	551	8,736
Wheat middlings	1960	10	2	430	2,364
	2010	10	2	490	4,222
Alfalfa hay	1960	2	1	101	499
	2010				
Fish meal	1960	6	3	714	4,620
	2010				
Poultry meat and bone meal	1960	191	71	6,472	31,165
	2010	121	45	4,605	42,437
Porcine meat and bone meal	1960	200	74	5,820	20,800
	2010	96	27	4,318	24,221
Ruminant meat and bone meal	1960	565	254	34,100	59,600
	2010	404	185	25,636	74,133
Poultry fat	1960	331	124	11,210	53,980
	2010	209	79	7,975	73,457
Porcine fat	1960	400	149	11,600	41,500
	2010	193	54	8,627	48,306
Ruminant fat	1960	1,136	511	68,468	119,788
	2010	812	371	51,546	148,951
Salt	1960	2	0	300	2,543
	2010	2	0	263	3,936
Limestone	1960	0	0	47	779
	2010	0	0	43	964
Calcium phosphate	1960	39	1	1,094	9,328
	2010	38	1	938	15,188

^1^e = equivalents. CDDGS = corn dried distillers grain with solubles.

As a result of both the differences in impacts attributable to feed inputs in 1960 compared with 2010 and changes in feed formulation over time (particularly decreased use of animal-derived meals and fats), a similar pattern was observed for pullet and layer feeds. Averaged across emission-related impact categories, impacts for a given quantity of feed produced in 2010 were reduced by 49% relative to 1960 for pullet feeds and reduced by 63% for layer feeds. In contrast, CED was 36 and 2% higher, respectively (Table 11).

**Table 11.** Life cycle impact assessment results for acidifying emissions, eutrophying emissions, greenhouse gas (GHG) emissions, and cumulative energy demand (CED) per metric ton of pullet and layer feeds produced in the United States in 1960 and 2010^1^

Feed and year	Acidifying emissions (kg of SO_2_-e)	Eutrophying emissions (kg of PO_4_-e)	GHG emissions (kg of CO_2_-e)	CED (MJ)
Pullet feed 1960	18.4	6.8	1,015	3,139
Pullet feed 2010	9.8	2.9	584	4,267
Reduction (%)	47	57	42	−36
Layer feed 1960	34.5	13.8	1,860	4,560
Layer feed 2010	12.5	4.4	782	4,632
Reduction (%)	64	68	58	−1.6

^1^e = equivalents.

#### Comparing Pullet Production Between 1960 and 2010.

**Figure 6.**
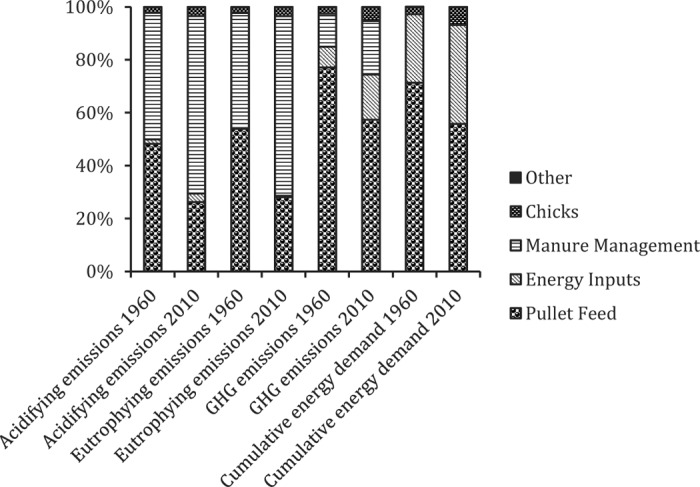
Contribution analysis for the life cycle impact assessment of pullets produced in the United States in 1960 compared with 2010. GHG = greenhouse gas.

Emissions-related impacts of pullet production in both 1960 and 2010 were largely driven by 2 factors—feed inputs and manure management (Figure 6). For CED, direct energy inputs to pullet houses rank second to feed inputs. However, the relative importance of these factors differed between 1960 and 2010. In 1960, feed inputs weighed most heavily across impact categories, particularly for GHG and CED. In 2010, manure management was the most important variable for acidifying and eutrophying emissions, due to decreased emissions associated with the production of feed inputs. The relative importance of direct energy inputs also increased in 2010, again due to the declining relevance of feed inputs as a result of changing feed composition (less animal-derived materials). Averaged across emissions-related impact categories, the environmental impact associated with pullet production was reduced by 56% in 2010 relative to 1960. Cumulative energy demand was also slightly reduced, at 9% (Table 12).

**Table 12.** Life cycle impacts assessment results for acidifying emissions, eutrophying emissions, greenhouse gas (GHG) emissions, and cumulative energy demand (CED) for 1,000 pullets and 1 t of eggs produced in the United States in 1960 and 2010

	Acidifying emissions (kg of SO_2_-e)		Eutrophying emissions (kg of PO_4_-e)		GHG emissions (kg of CO_2_-e)		CED (MJ)	
Item	Pullets	Eggs	Pullets	Eggs	Pullets	Eggs	Pullets	Eggs
Year								
1960	390	200	129	70	13,458	7,230	45	18
2010	196	70	54	20	5,404	2,080	41	12
Reduction (%)	50	65	58	71	60	71	9	31

#### Comparing Egg Production Between 1960 and 2010.

**Figure 7.**
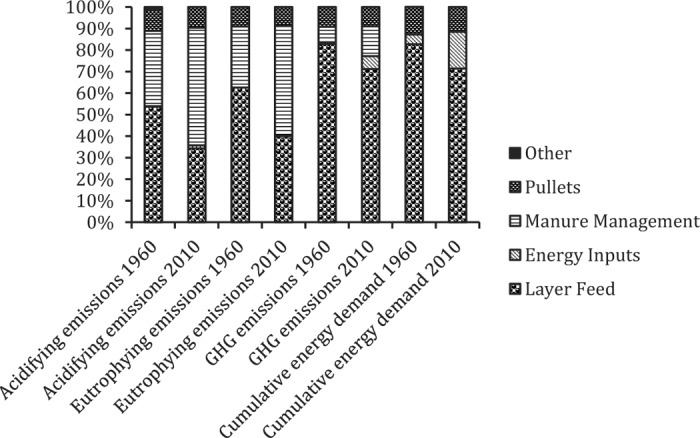
Contribution analysis for the life cycle impact assessment of eggs produced in the United States in 1960 compared with 2010. GHG = greenhouse gas.

The distribution of impacts for egg production was very similar to that of pullet production for both 1960 and 2010. In 2010, manure management replaced feed inputs as the largest source of acidifying and eutrophying emissions (despite substantially lower losses of N and P per quantity of eggs produced), whereas feed remained the dominant (although smaller) contributor to both GHG emissions and CED. These changes reflected both changing feed composition and improved feed use efficiency. Pullet production contributed approximately 10% to emissions-related impacts in both 1960 and 2010, and slightly more for CED (Figure 7). In general, direct energy inputs were of lesser importance. Overall, emissions-related impacts of egg production in 2010 were reduced by 69% relative to 1960, whereas CED was reduced by 31% (Table 12).

#### Analysis of Drivers of Observed Differences in Impacts Between 1960 and 2010.

Applying 2010 background system submodels in the 1960 egg production model, we estimated that 27 to 30% of the observed differences in acidifying, eutrophying, and GHG emissions were attributable to changes in the efficiencies of background systems such as fertilizer and feed input production, and transport modes. These outweighed the declining EROI for primary energy carriers in these impact categories. For CED, however, applying 2010 energy carriers to the 1960 model resulted in 35% higher impacts in this category (Table 13).

**Table 13.** Proportion (in %) of changes in the environmental footprint [acidifying emissions, eutrophying emissions, greenhouse gas (GHG) emissions, and cumulative energy demand (CED)] of egg production in the United States in 2010 compared with 1960 attributable to changes in background systems, feed composition, or bird performance due to improved husbandry and genetics

Footprint change attributable to changes in	Acidifying emissions	Eutrophying emissions	GHG emissions	CED
Background systems (%)	27	30	28	−116
Feed composition (%)	30	35	44	93
Bird performance (%)	43	35	28	123

Using both 2010 background system models and feed composition in the 1960 egg production model, we further estimated that changes in feed composition over time accounted for 30% of the observed decline in acidifying emissions for egg production in 2010, 35% for eutrophying emissions, and 44% for GHG emissions. The remaining proportion of observed decline was attributable to improved bird performance over the 50-yr interval (e.g., better feed efficiency, lower mortality rate): 43% for acidifying emissions, 35% for eutrophying emissions, and 28% for GHG emissions. Despite declining EROI, CED in 2010 was only 30% that of 1960, due to a combination of changing feed composition and improved bird production practices (Table 13).

#### Comparison with Other Studies.

A limited number of temporal analyses of the environmental impacts of animal production are available. Capper et al. (2009) and Capper (2011) evaluated changes in the environmental performance of beef production in 1977 versus 2007, and dairy production in 1944 versus 2007. Considerable gains in the efficiency of resource utilization (69.9% of animals, 81.4% of feedstuffs, 87.9% of the water, and 67.0% of the land required) per kilogram of beef produced in 2007 compared with 1977, and commensurate decreases (16.3%) in associated GHG emissions, were documented. Similar gains in resource efficiency were estimated for dairy (21% of animals, 23% of feedstuffs, 35% of the water, and only 10% of the land per kg of milk produced in 2007 compared with 1944), whereas GHG emissions were 37% of 1944 levels.

It should be noted that these studies (nor those discussed below) did not take into account changes in the resource efficiencies of background systems, hence are likely quite conservative. Our estimates of the scale of resource efficiencies and emission reductions for egg production between 1960 and 2010 are, nonetheless, of a comparable magnitude. In Canada, Vergé et al. (2009) calculated direct GHG emissions from layer facilities along with crops used to produce layer feeds in 1981 compared with 2006. Indirect supply chain emissions were not considered; hence, the study results are not directly comparable with those presented in the current analysis. Still, it is interesting to note that these authors found that the GHG intensity of egg production decreased from 1.9 kg of CO_2_ equivalents/dozen eggs in 1981 to 1.76 kg of CO_2_ equivalents/dozen eggs in 2006, an approximately 7% reduction over the 25-yr interval. Cederberg et al. (2009) compared GHG emissions from Swedish livestock production in 1990 and 2005 for pork, poultry meat, beef, milk, and eggs. They found that the carbon footprint of pork production decreased from 4 to 3.4 kg of CO_2_ equivalents/kg over the 15-yr interval, emissions for poultry meat decreased from 2.5 to 1.9 kg of CO_2_ equivalents/kg, and emissions for milk from 1.27 to 1 kg of CO_2_ equivalents/kg. Emissions for beef production increased from 18 to 19.8 kg of CO_2_ equivalents/kg. Emissions from egg production remained unchanged at 1.4 kg of CO_2_ equivalents/kg over this interval. This latter finding was largely attributable to 2 factors: a) the phasing out of animal by-products in feeds as a result of bovine spongiform encephalopathy (**BSE**) concerns, and b) the use of economic allocation in modeling. Here, despite efficiency gains in the sector, the allocation strategy resulted in a study outcome suggesting no net gains in environmental performance.

To date, no other estimates for the life cycle impacts of contemporary national average US egg production are available. Pelletier et al. (2013) previously modeled egg production in Iowa using the same modeling approach as applied in this analysis. In the Iowa study, the authors did not identify the precise source (ruminant, swine, or poultry) of animal-derived meals and fats. However, they estimated that GHG emissions ranged from 2.0 kg of CO_2_ equivalents (assuming 100% of the animal-derived products were of poultry origin) to 5.0 kg of CO_2_ equivalents per kg of eggs produced (assuming 100% of the animal-derived products were of ruminant origin).

Several studies are available, however, that report environmental performance for egg production supply chains in other countries (Table 14). Although direct comparisons between studies are problematic due to frequent differences in modeling assumptions (e.g., system boundaries for the studies, data sources, allocation strategies, and so on), it is nonetheless interesting to consider the range of reported impacts relative to those of the current study.

**Table 14.** Reported life cycle impacts for acidifying emissions, eutrophying emissions, greenhouse gas (GHG) emissions, and cumulative energy demand (CED) per kilogram of eggs produced in different countries^1^

Study	Acidifying emissions (g of SO_2_-e)	Eutrophying emissions (g of PO_4_-e)	GHG emissions (kg of CO_2_-e)	CED (MJ)
US average (this study)	70	20	2.1	12.3
United Kingdom^2^	53	77	2.9	16.8
The Netherlands^3^	32	25	3.9	—
Sweden^4^	—	—	1.4	—
Canada^5^	—	—	2.5	—
Australia^6^	—	—	1.4	—

^1^e = equivalents.

^2^Leinonen et al. (2012).

^3^Mollenhorst et al. (2006).

^4^Cederberg et al. (2009).

^5^Vergé et al. (2009).

^6^Wiedemann and McGahan (2011).

In broad strokes, the distribution of impacts along contemporary US egg supply chains seems to be in general agreement with similar, previously reported LCA research of intensive, cage egg production systems elsewhere (Mollenhorst et al., 2006; Cederberg et al., 2009; Vergé et al., 2009; Wiedemann and McGahan, 2011; Leinonen et al., 2012). In a study examining the social, economic, and ecological dimensions of egg production by housing system in the Netherlands, Mollenhorst et al. (2006) used LCA as a basis for comparing performance in the environmental domain. Conventional cage production was found to perform better according to the environmental LCA variables considered, but the aviary system performed better according to the economic and animal welfare measures employed. In Australia, Wiedemann and McGahan (2011) used a life cycle approach to evaluate GHG emissions, energy, and water use in egg production by housing system. Here, activity data were collected from 4 farms in eastern Australia. Cage systems were found to outperform free-range systems. Estimated impacts overall were low compared with results from most European studies. More recently, Leinonen et al. (2012) used top-down estimates of average UK production conditions in a standard, environmental LCA approach to characterize environmental performance for egg production in cage, barn, free-range, and organic systems. They reported highest impacts for organic production and lowest for cage production, largely due to differences in productivity (i.e., higher feed consumption and number of birds required per unit of egg production in the organic system). Feed production supply chains were the dominant contributor to GHG emissions (64–72%) and CED (54–75%). Similar to our study, energy use in housing systems was the second most important factor for the overall energy intensity of egg production. Manure management contributed most to acidifying and eutrophying emissions.

Where estimated impacts in these other studies (Table 14) are low compared with those of the present analysis, this is typically either because animal by-products are not allowed for use in animal feeds in the countries of concern (e.g., the Swedish study by Cederberg et al. for the 2005 system modeled), or because they were not included in the modeled feeds at all, whether or not they are actually used (e.g., the Australian study by Wiedemann and McGahan). In the latter study, the authors also point toward the low input nature of Australian grain production (compared with European norms) as an important factor influencing their reported outcomes. Considering the study of egg production in Sweden in 1995 compared with 2005 (Cederberg et al., 2009), the reduction in use of animal by-product due to legislative changes in response to BSE concerns over this interval in fact *negatively* affected performance in 2005 due to the use of economic allocation in this study. This is contrary to the results of the current analysis, which showed an improved environmental performance over time by reducing the amount of animal-derived materials used in poultry diets. In light of the resource and emissions intensity of producing livestock (along with the livestock processing coproducts used in animal feeds), the analytical approach of the current study better reflects the actual environmental costs of producing feed inputs for egg production, regardless of the economic value of such materials.

To put the GHG intensity of contemporary US egg production in perspective, the following comparison is provided. Using the same methods, Pelletier et al. (2010a) recently estimated the GHG emissions to be 3 kg of CO_2_ equivalents per kg of live weight pork produced in the Midwestern United States. For conventional, feedlot beef production, the estimated GHG emissions were 14.5 kg of CO_2_ equivalents per kg of live weight produced (Pelletier et al., 2010b). Similarly, an earlier study of US broiler production (Pelletier, 2008) revealed an estimated GHG emission of 1.7 kg of CO_2_ equivalents per kg of live weight produced. Here, we estimated a GHG intensity of 2.1 kg of CO_2_ equivalents per kg of eggs produced in the continental United States in 2010, compared with 7.2 kg of CO_2_ equivalents per kg of eggs produced in 1960.

Making a similar comparison on the basis of protein, the GHG intensity, expressed as kilograms of CO_2_ equivalent emissions per kilogram of protein produced, is 19.1 for contemporary (2010) US egg protein (raw, from whole eggs), compared with 11.5 for broiler protein, 17.6 for pig protein, and 78.4 for beef protein.

Clearly, the US egg sector has made significant strides in improving resource utilization efficiency and reducing environmental impacts per unit of production since the 1960s. It is equally or more important to consider the extent to which such improvements have affected the total environmental footprint. The total US table egg production in 1960 was 59.8 billion eggs compared with 77.8 billion in 2010 (NASS, 2012), an increase of approximately 30%. Despite the substantial increase in production volume, the total CED in the US egg industry decreased by 13%, whereas total GHG emissions declined by 63%, total acidifying emissions by 54%, and total eutrophying emissions by 63%.

### Conclusions and Recommendations

The distribution and magnitude of environmental impacts for US egg production in 2010 and 1960 were analyzed using LCA. The results clearly showed remarkable resource efficiency and environmental performance gains, both per unit production and in aggregate, achieved by the industry over the past 50 yr. The primary influencing factors and their relative contributions to the reductions in environmental footprint were elucidated. Specific insights and key findings are as follows.

From a supply chain management perspective, the key to improving environmental performance in egg production has been and will continue to be efforts to maximize feed efficiency. Feed conversion (feed to egg ratio) for egg production improved from 3.44 in 1960 to 1.98 in 2010, a 42% improvement. Achieving feed efficiencies comparable with the best-performing contemporary facilities (reported feed conversion ranged from 1.76 to 2.32) industry-wide would further reduce aggregate impacts.

Changing feed composition has also played an important role in reducing impacts. This is especially the case with both reduction in the total amount of animal-derived materials used and increased use of porcine and poultry materials in place of ruminant materials. The concept of least-environmental cost feed sourcing is therefore of particular relevance for additional targeted performance improvements for the egg industry. It is recommended that similar biophysical accounting methods to those applied in the current study be used to model potential alternative feed input supply chains to ensure methodological consistency and comparability with the present analysis.

Nitrogen losses from poultry manure are the second largest contributor to acidifying and eutrophying emissions, as well as a nontrivial contributor to GHG emissions for both pullet and layer facilities. Moreover, upstream impacts of N fertilizer production and use are a primary determinant of feed input-related impacts. Feed formulation, breeding, and manure management strategies for optimal N use efficiencies are therefore powerful tools in supply chain environmental management.

The benchmarks reported here, as well as the reported ranges for resource use and production efficiencies in otherwise similar production facilities, provide an excellent reference point for industry-led initiatives to further improve the environmental performance of US egg production.
